# Effect of Platelet-Rich Fibrin Coating on Secondary Stability of Dental Implants: A Systematic Review and Meta-Analysis

**DOI:** 10.7759/cureus.75166

**Published:** 2024-12-05

**Authors:** Shivi Khattri, Mayur Kaushik, Nitin Tomar, Sameer Ahmed, Nazar Rana, Mehvish Khan, Soundarya Singh, Roopse Singh

**Affiliations:** 1 Periodontology, Subharti Dental College, Swami Vivekanand Subharti University, Meerut, IND

**Keywords:** endosseous dental implants, implant surface modification, platelet-rich fibrin (prf), surface biomodification, systematic review and meta analysis

## Abstract

A systematic review of the effect of platelet-rich fibrin (PRF) on the secondary stability of delayed dental implants is lacking. This systematic review and meta-analysis aims to evaluate if PRF's application on delayed implant placement enhances secondary stability. A comprehensive search was done on Pubmed, Cochrane Library, Embase, and Scopus databases to retrieve the records. Only randomized controlled trials (RCTs) or controlled clinical trials (CCTs) were included in the review. The outcome was secondary stability values measured in the implant stability quotient (ISQ). The meta-analysis was performed using a random effects model with 95% confidence intervals (CIs). We assessed the certainty of evidence using GRADEpro. We included 12 trials conducted worldwide involving 456 dental implants installed. Six studies showed a high risk of bias, whereas three had a low risk of bias, and three had an unclear risk of bias. One trial did not contribute to meta-analysis. Certainty of evidence was assessed for only one comparison, which reported follow-up at 12 weeks. Implant + PRF versus Implant + no PRF: the evidence for the secondary stability of implant at four weeks (Mean difference (MD) 3.34, 95% CI 2.24 to 4.43; implants = 302; studies = 8; I2 = 0%); at six weeks (MD 2.53, 95% CI 0.85 to 4.20; implants = 146; studies = 3; I2 = 0%) and at ≥12 weeks (MD 3.37, 95% CI 0.69 to 6.06; participants = 162; studies = 4; I2 = 17%) was of low certainty.

There is low certainty of evidence (≥12 weeks follow-up) for implant stability by PRF coating during installation to confirm if it can be useful for the clinicians during the delayed dental implant placement. Well-planned RCTs need to be conducted with longer follow-ups of ≥12 weeks to strengthen the evidence.

## Introduction and background

Tooth loss has become a major problem as it affects a person's quality of life and well-being [1]. Therefore, replacing a missing tooth is imperative. There are many treatment options for this. Dental implants are a newer treatment modality that replaces the missing tooth and helps prevent the conservation of adjacent teeth. Dental implants also improve the patient's function, aesthetics, and self-confidence. 

There have been constant efforts to improve the success of the implants. For a dental implant to be successful, it should fulfill the criteria [2]. However, with the advent of new materials, the success criteria have been modified per the requirements [3]. The most accepted success criteria for dental implants were given by Albrektsson et al. [2], which state the following for the implant success: a) Individual unattached implant that is immobile when tested clinically; b) Radiography that does not demonstrate evidence of peri-implant radiolucency; c) Bone loss that is less than 0.2 mm annually after the implant′s first year of service; d) No persistent pain, discomfort or infection; e) By these criteria, a success rate of 85% at the end of a 5 year observation period and 80% at the end of 10 years are minimum levels for success [4].

Various methods have been tried to improve the implant survival rate and success. This includes: a) Physical modification (at macro, micro, and nano level); b) Biological modification.

Physical modification of dental implant surfaces is done at macro, micro, and nano levels [5]. Macro-level modifications are done by modifying visible geometry ranging on a millimeter scale. These modifications alter the implant shape [6] and thread geometry (thread shape, thread pitch, face angle, thread depth, helix angle, crestal module). This is done to increase the macro-roughness, thereby enhancing the mechanical interlocking between the implant surface and bone. This further augments the primary and secondary stability of the implant [7,8]. Micro-level roughness refers to the modification of the implant surface by the process of machining, acid-etching, anodization, sand-blasting, grit-blasting, and other coating procedures [9]. These processes increase the surface area by forming pits, grooves, and protrusions [9]. The roughness thus created orchestrates the process of osseointegration by increasing the bone-implant-contact (BIC) [10]. The roughness is of the order of 1-100µ [9]. These procedures create roughness, but they are not purely physical procedures, as the use of chemical substances is done to alter the physical structure of the dental implant surface. So, they are ideally the blend of physical and chemical procedures. Nano level modifications are of the order 1-100nm. Nano-roughness not only influences the topography but also alters the surface chemistry of the dental implant surface [11,12]. It can be done by three methods: a) molecular self-assembly, b) physical method of compaction of nano-particles, and c) chemical treatment of surfaces [11,13].

Biological modification can be done using: a) Platelet-rich plasma (PRP) and platelet-rich fibrin (PRF) [14]; b) Extracellular matrix (ECM) [15]; c) Peptides [16,17]; d) Growth factors [18]; e) Drugs [19]; f) Combination [14]

The biological methods of making the implant surface "bioactive" have been tried in many studies. However, most of the studies are either in vitro or on animal models [14-19]. However, there have been few studies in humans as well. Regardless of the microtopographic nature of the dental implant surface, it is well known that the initial interaction of blood proteins with the implant surface immediately after its placement is the determining factor for the succeeding wound-healing biological events [20]. Thus, the platelet concentrates can be used to coat the implant surfaces during the initial stages of osseointegration. This, in turn, influences the upcoming healing events [21]. It can be postulated that this surface bio-modification using autologous blood products, namely PRF, may act as a "biomimetic" coating on the implant surface. This enhances the biocompatibility of the dental implant by providing extracellular matrix proteins and growth factors from the neighboring areas of the surgical site. Further, it improves the rate and extent of bone formation during the later stages of osseointegration [22]. So, this review aims to evaluate the efficacy of PRF application on the implant surface on the implant's secondary stability.

## Review

Methods

The protocol has been registered in the International Prospective Register of Systematic Reviews (PROSPERO #CRD42024547926). Data were assessed by following the Preferred reporting items for systematic reviews and meta-analyses (PRISMA) guidelines [23].

The PICO question for the present review is:

"In patients getting delayed dental implants, is the PRF coating on dental implant surface effective compared to no coating in terms of secondary stability?"

Inclusion criteria: Randomized controlled trials (RCTs) and Clinical Controlled trials (CCTs), including systemically healthy individuals who require dental implant placement.

Exclusion criteria: Case-reports, case-series, and quasi-randomized trials were all excluded.

Electronic searches of MED-LINE, SCOPUS, and Cochrane Library databases were conducted in March 2024 for publications that investigated the effect of extemporaneous implant surface biomodification. In MEDLINE, the Medical Subject Heading term "implant surface biomodification" with the Boolean operator OR was linked to the terms "chemical modification of implant surface", "implant surface modification" or "dental implant surface modification" OR "PRF coating on implant" OR "platelet-rich fibrin coating on implant". Similar search strategies were used to retrieve records from other databases. Keywords were in English. No other language or date restrictions were applied. Two pairs of reviewers (SK & MK and SA and NT) appraised obtained titles and abstracts. Full-text copies of the remaining potential citations were obtained, and the following inclusion criteria were applied: randomized controlled trials (RCTs)/clinical controlled trials (CCTs) evaluating extemporaneous biological modification by PRF of dental implant surface (i.e., at the time of implant placement) as compared to no biomodification of the same. Studies carrying out immediate implant placement were also excluded. Also, a manual search was conducted to ensure that no trial was left from getting included in the review. Articles meeting the inclusion criteria were combined with the articles obtained from the electronic search. Each reviewer was then unmasked to the other's progress, and a consensus was reached regarding any citations selected by only one reviewer. 

The full-text articles obtained were carefully scanned and read to extract the data. Two pairs of reviewers did data extraction, NT & SA and SS & MS. The following data was extracted from each article: 1) Type of study; 2) No. of participants; 3) Age; 4) Sample size; 5) Intervention used; 6) Outcomes assessed; 7) Follow-up

The extracted data is shown in Table 1. Two investigators (RS, MS) independently assessed the risk of bias using a quality assessment checklist [24]. Any disagreement between the two authors was resolved by discussion. When resolution was not possible, we consulted an arbiter (SK).

Funnel plots were used to assess the publication bias if the number of studies included in the meta-analysis was more than 10. Also, sensitivity analysis was performed to assess the contribution of each study to the overall estimate. 

Results

**Figure 1:**
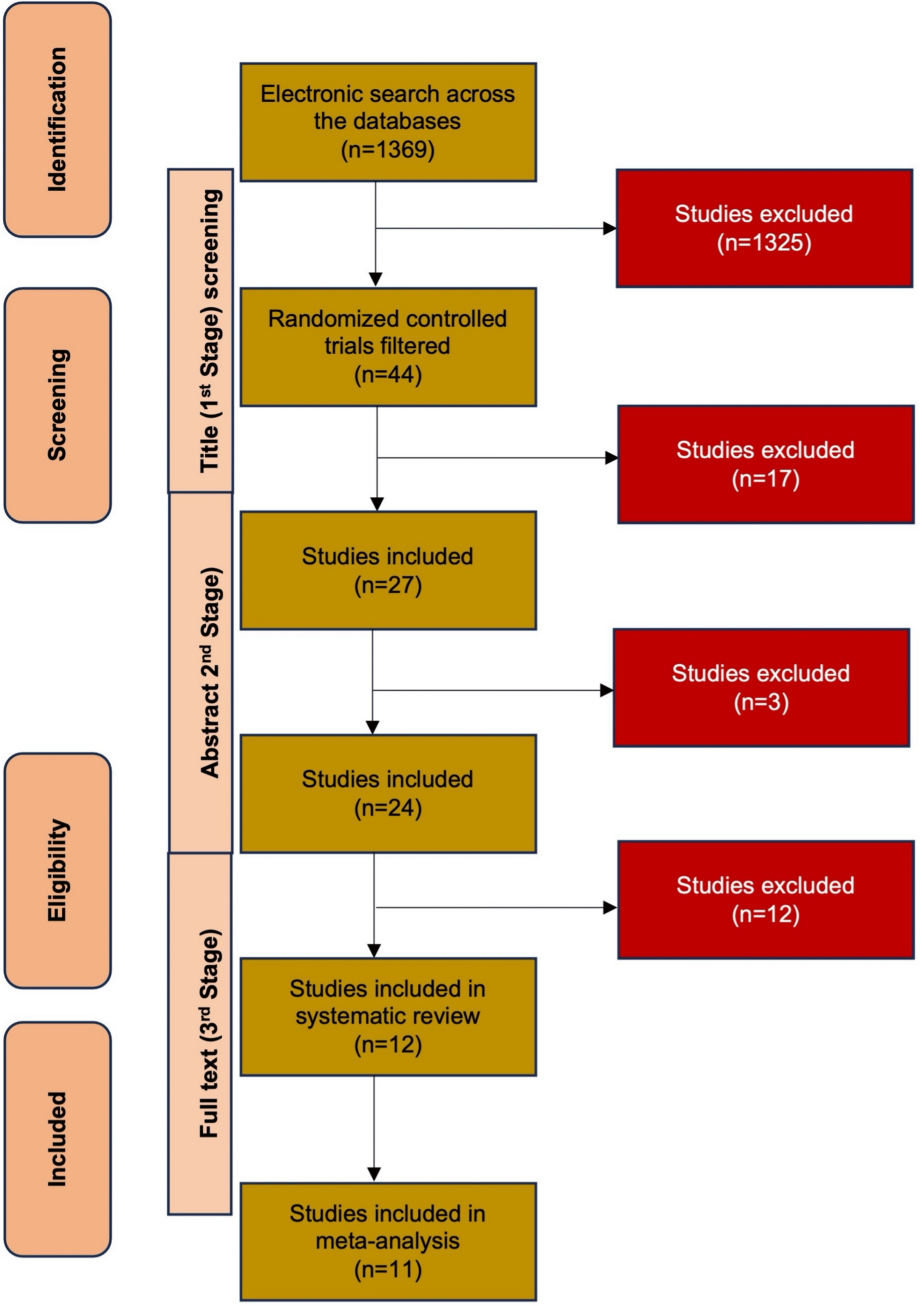
PRISMA flowchart n: No of records; PRISMA: Preferred Reporting Items for Systematic Reviews and Meta-Analyses

With the search strategy mentioned above, 1368 records were obtained. Out of these, 44 studies were RCTs/CCTs. These studies underwent three-stage screening. In the first stage, studies were screened for their titles. This yielded 27 studies, and 17 studies were excluded. The second screening stage included abstract screening, and 24 studies were included. The final screening was carried out through intensive full-text screening of the 24 studies. This was further reduced to only 12 studies [25-36], which matched the inclusion criteria. All the studies had compared implant installation with and without coating of PRF. So, all the included studies were analyzed in the systematic review. Out of 12 studies, only 11 measured the outcome (ISQ value) were included in the meta-analysis (Figure 1). The characteristics of 12 included [25-36] and excluded studies [37-48] are shown in Tables 1-2 respectively.

**Table 1:** Characteristics of included studies CCT: Controlled clinical trials; RCT: Randomized controlled trials; CGF: Concentrated growth factor; PRF: Platelet-rich fibrin; ISQ: Implant stability quotient; CBL: Crestal bone level; MD: Mean difference; I: Intervention; C: Control

S.No	Study ID	Type of Study	Age (years)	Sample size (n) (no. of implants)		Intervention	Outcomes assessed	Time of measurement of secondary stability	Key findings
				I	C				
1	Alhussaini et al. 2019 [25]	CCT, Parallel	I: 49.4; C:48.6	17	32	I: implant coated with PRF; C: implant only	ISQvalues	6 week, 12 week	At 6 weeks, the ISQ values decreased for both groups. At 12 weeks, the values were higher for the PRF group but were not statistically significant.
2	Anapu et al. 2024 [26]	CCT	34.4	13	13	I: implant coated with PRF; C: implant only	ISQ values	6 month	Significant secondary stability with PRF.
3	Boora et al. 2015 [27]	RCT, Parallel	24.6	10	10	I: implant coated with PRF; C: implant only	CBL	3 month	The amount of CBL changes in the study group had a statistically significant lesser mean value than in the control group.
4	de Oliveira et al. 2022 [28]	Split-mouth RCT	67.73	15	15	I: implant coated with PRF; C: implant only	ISQ values	1-year	ISQ values in both groups increased, but there was no statistically significant difference.
5	Güvenç et al. 2022 [29]	RCT, Parallel	25-67	20	20	I: implant coated with PRF; C: implant only	ISQ values	4 week	The study group showed an increase in ISQ values in the fourth week, and were statistically significant.
6	Kapoor et al. 2022 [30]	Split mouth RCT, Parallel	42	30	30	I: implant coated with PRF; C: implant only	ISQ values	1 week, 1 month, 3 month	On intergroup comparison, statistically significant values of ISQ in the intervention group at 1 week and 1 month. but statistically non-significant values at 3 month.
7	Naeimi et al. 2023 [31]	Split-mouth RCT	48.93	14	14	I: implant coated with PRF; C: implant only	ISQ values	1 week, 2 week, 4 week, 6 week, 8 week, 12 week	There is no statistically significant difference in ISQ values over time between the two groups.
8	Oncu et al. 2015 [32]	RCT	44.2	10	10	I: implant coated with PRF; C: implant only	ISQ values	4 week	Enhanced stability of the implants with PRF.
9	Ozveri-Kuuncu et al. 2020 [33]	RCT	67	12	12	I: implant coated with PRF; C: implant only	ISQ values	1 week, 2 week, 4 week	The difference in the ISQ values of the two groups was not statistically significant.
10	Pirpir et al. 2017 [34]	Split-mouth RCT	44	20	20	I: implant coated with CGF membrane; C: implant only	ISQ values	4 week	The ISQ values in the intervention group were notably higher, and implant stability improved.
11	Tabrizi et al. 2018 [35]	Split-mouth CCT	39.6	20	20	I: implant coated with PRF; C: implant only	ISQ values	2 week, 4 week, 6 week	Higher ISQ values in the intervention group. Implant stability may increase in the PRF group.
12	Torkzaban et al. 2018 [36]	RCT	-	25	25	I: implant coated with PRF; C: implant only	ISQ values	1 week, 4 week	Significant increase in ISQ values in the intervention group as compared to the control group.

**Table 2:** Characteristics of excluded studies PRP: Platelet-rich plasma; PRF: Platelet-rich fibrin

S.No	Study ID	Reason for exclusion
1.	Diana et al. 2018 [37]	Immediate implant placement was done.
2.	Dominiak et al. 2021 [38]	Sinus lift has been done.
3.	Elbrashy et al. 2022 [39]	Immediate implant placement was done.
4.	Elsheikh et al. 2023 [40]	Immediate implant placement was done.
5.	Ergun et al. 2013 [41]	PRPwas used in the study.
6.	Gaur at al. 2022 [42]	Immediate implant placement was done.
7.	Hartlev et al. 2021 [43]	Use of autogenous graft with PRF.
8.	Khan et al. 2018 [44]	Immediate implant placement was done.
9.	Khan et al. 2021 [45]	PRP was used in the study.
10.	Monov et al. 2005 [46]	PRP was used in the study.
11.	Oncu et al. 2016 [47]	Animal study.
12.	Oncu et al. 2019 [48]	Immediate implant placement was done.

**Figure 2:**
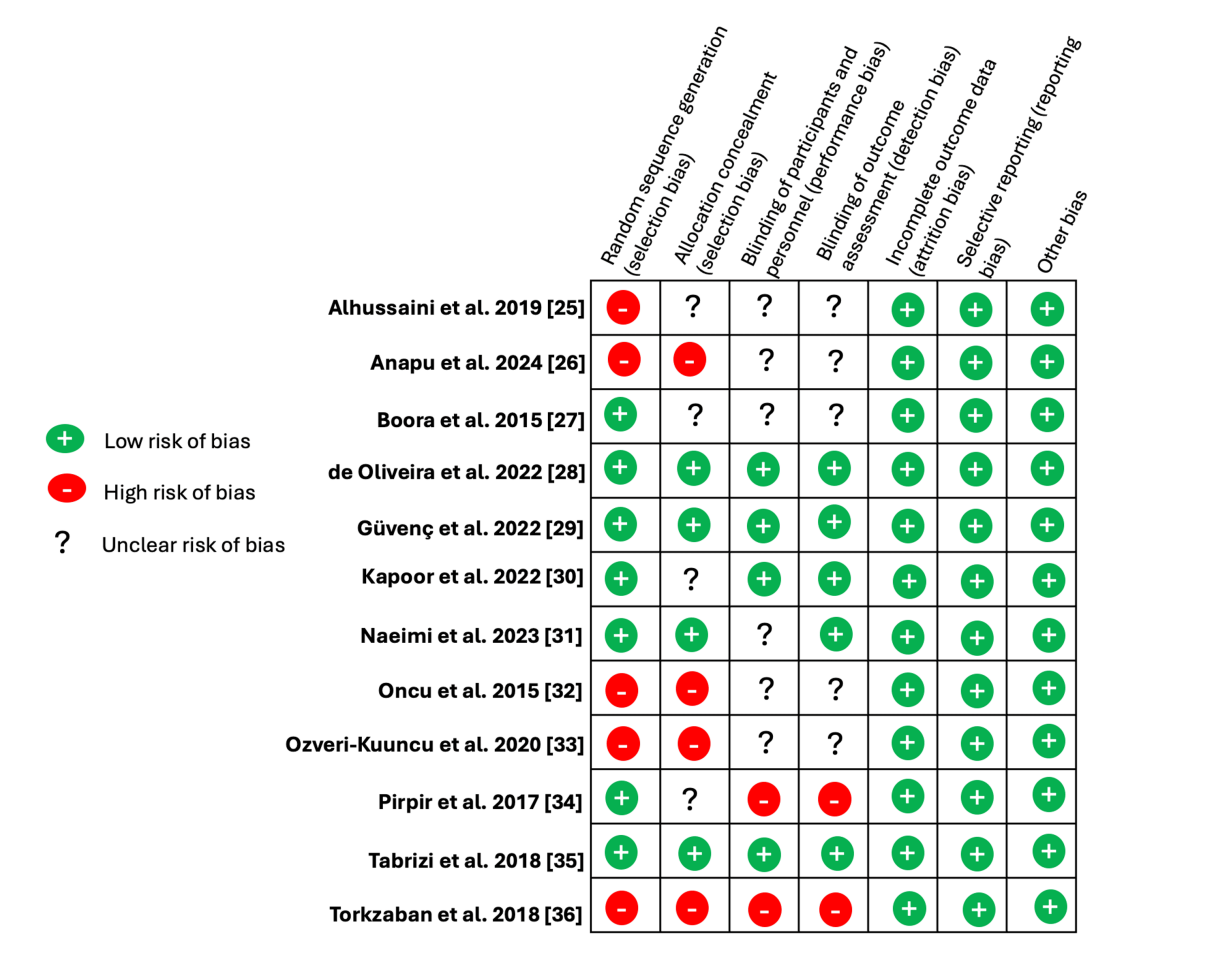
Risk of bias summary: Review authors' judgments about each risk of bias item for each included study.

**Figure 3:**
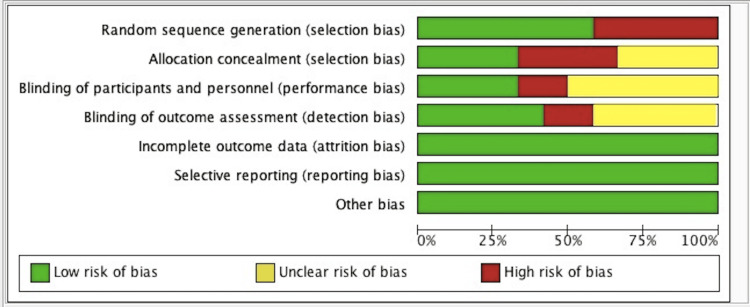
Risk of bias graph: Review authors' judgments about each risk of bias item presented as percentages across all included studies.

The quality of the studies as assessed by the risk of bias tool [24] shown in Figures 2-3 were considered as having "low risk", "unclear risk" or "high risk" based on the assessment of the following biases: a) selection bias; b) performance bias; c) detection bias; d) reporting bias; and e) attrition bias. Five studies [25,26,32,33,36] showed high risk in selection bias assessment as randomization was either not done or it wasn't clear if randomization was done properly. Four studies [26,32,33,36] showed high risk of bias in allocation concealment. Two studies showed high risk of detection bias [34,36].

**Figure 4:**
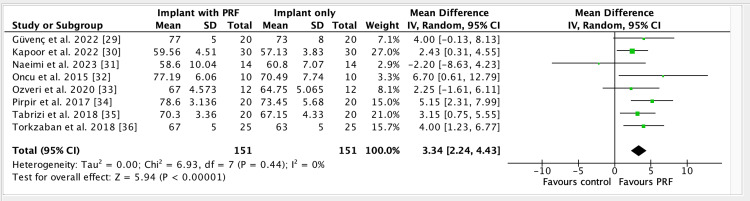
Forest plot of comparison: Implant + PRF versus Implant + no PRF, outcome: ISQ values at four-week follow-up PRF: Platelet-rich fibrin; SD: Standard deviation; CI: Confidence interval; IV: Inverse variance; df: Degree of freedom; I2: Statistical tool for measuring inconsistency/heterogeneity; ISQ: Implant stability quotient

**Figure 5:**
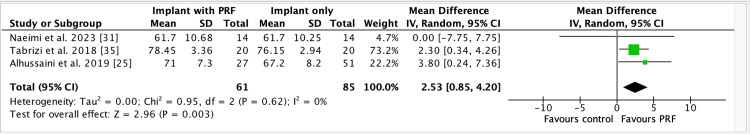
Forest plot of comparison: Implant + PRF versus Implant + no PRF, outcome: ISQ values at six-week follow-up PRF: Platelet-rich fibrin; SD: Standard deviation; CI: Confidence interval; IV: Inverse variance; df: Degree of freedom; I2: Statistical tool for measuring inconsistency/heterogeneity; ISQ: Implant stability quotient

**Figure 6:**
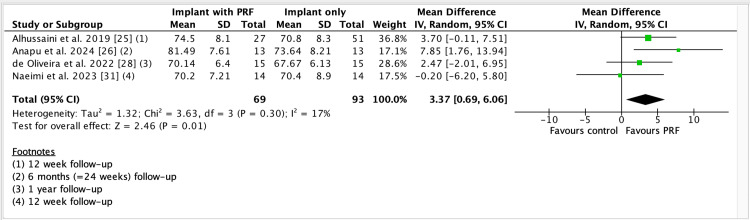
Forest plot of comparison: Implant + PRF versus Implant + no PRF, outcome: ISQ values at >12 week follow-up PRF: Platelet-rich fibrin; SD: Standard deviation; CI: Confidence interval; IV: Inverse variance; df: Degree of freedom; I2: Statistical tool for measuring inconsistency/heterogeneity; ISQ: Implant stability quotient

We included 12 trials involving 456 dental implants installed. One trial did not contribute to meta-analysis. Secondary stability was assessed at four, six, and 12 weeks. MD for ISQs at four weeks (Fig 4) was 3.34 (95% CI 2.24 to 4.43; implants = 302; studies = 8; I2 = 0%, indicating higher stability of dental implants with the use of PRF. The mean difference (MD) at six weeks (Fig 5) was 2.53 (95% CI 0.85 to 4.20; implants = 146; studies = 3; I2 = 0%) also indicates increased stability of dental implants when PRF was used. MD at 12 weeks (Fig 6) was 3.37, 95% CI 0.69 to 6.06; participants = 162; studies = 4; I2 = 17%).

Certainty of evidence was assessed (using GRADEpro) for only one comparison, which reported follow-up at ≥12 weeks. Implant + PRF versus Implant + no PRF: The evidence for the secondary stability of implant at ≥12 weeks was of low certainty. The main reason for downgrading the evidence was because of non-randomized studies.

Funnel plot asymmetry was not done as the number of studies included in the meta-analysis was less than 10. Sensitivity analyses were done by excluding each study, but that didn't affect the overall estimate. Hence, the analysis was shown without applying the sensitivity analysis, i.e., without omission of any study.

Discussion

The measurement of resonance frequency analysis (RFA) can provide clinically appropriate details of the condition of the implant-bone interface at any stage during treatment or at follow-up. RFA also helps determine the status of implant stability in terms of the stiffness of the implant-bone interface. The values of RFA are also influenced by healing time, bone density, and the amount of exposed implant height above the alveolar crest [49]. Implant osseointegration can be enhanced in two different ways, which include modifying its topography and physicochemistry [50]. When the PRF is applied to the dental implant surface, it seems to enhance implant stability regarding ISQ values.

Lyris et al. [51] conducted a similar systematic review to evaluate the impact of leukocyte- and platelet-rich fibrin (L-PRF) application in an implant bed before implant placement. It evaluated the implant stability by measuring ISQ values. A total of four RCTs were included for data extraction. The studies were analyzed to have moderate to unclear risk of bias. Meta-analysis was performed to assess the effect of L-PRF on implant stability immediately post-insertion in three studies, after one week from the implant placement in three studies, and after four weeks for all the included studies. The results for both follow-up intervals favor the use of L-PRF. However, immediately after the implant placement, statistical differences were not significant among the groups. It suggests that L-PRF enhances secondary implant stability. However, it should be clinically correlated so one can be sure of its positive outcome.

In their study, Ivanovski et al. [22] stated that there is limited evidence of the clinical benefits of adding the liquid form of L-PRF to any implant surface. When L-PRF as a membrane is used in the osteotomy site, however, it may bring about positive clinical effects during initial healing by enhancing early implant stability and minimizing marginal bone loss, although no positive longer-term effects were observed. Therefore, while drawing conclusions from these studies, one should be very cautious, as the methodology had limitations.

Previous studies have reported various centrifugation protocols that affect the concentration of platelets and, thus, the amount of growth factors. These variations across the studies are inevitable, as standardizing the protocol for PRF might be challenging because of the initial manufacture differences in the PRF centrifugation apparatus.

Nevertheless, the implant macro-design, diameter, length, and surface characteristics also significantly influence implant stability. Bone quality also influences implant stability. All these factors can cause inherent variations in the results.

## Conclusions

PRF can be used as an adjunct during implant placement as it enhances implant stability because the overall pooled estimate favors implant placement with PRF. But as far as certainty of evidence is concerned, it came out to be low. Future studies should target how PRF influences and interacts with the implant surface. Also, controlled preclinical and clinical studies should be designed to utilize standardized PRF preparation and application protocols.
